# 1,5-Bis[2,6-bis­(2,4,6-triisopropyl­phen­yl)phen­yl]-2,3,4,6,7-penta­tellura-1,5-di­stannabicyclo­[3.1.1]hepta­ne

**DOI:** 10.1107/S1600536810025729

**Published:** 2010-07-03

**Authors:** Masaichi Saito, Hizuru Hashimoto, Tomoyuki Tajima

**Affiliations:** a Department of Chemistry, Graduate School of Science and Engineering, Saitama University Shimo-okubo, Saitama-city, Saitama 338-8570, Japan

## Abstract

The title compound, [Sn_2_(C_72_H_98_)Te_2_(Te_3_)], has a cage-like structure with bulky aryl substituents on the Sn atoms. The mol­ecule sits over a crystallographic twofold axis, and hence the asymmetric unit consists of one half-mol­ecule. Due to the twofold axis, the tritelluride part has a 1:1 disorder. One of the six-membered rings has a boat conformation, whereas the other has a chair conformation. The ditelluradistannane ring has a bent structure, with a dihedral angle of 32.89 (2)° between the two Te—Sn—Te planes.

## Related literature

For related structures, see: Sladky *et al.* (1985 ▶), Hamor *et al.* (1986 ▶); Herberhold *et al.* (1990 ▶); Beckmann *et al.* (2009 ▶). For mol­ecular structures of polythia- and polyselena­dimetalla­bicyclo­[*k.l.m*]alkanes, see: Yoshida *et al.* (1992 ▶); Ando, Choi *et al.* (1994 ▶); Ando, Kabe *et al.* (1994 ▶); Ando *et al.* (1995 ▶); Choi *et al.* (1995 ▶, 1996 ▶, 1997 ▶). For other related structures, see: Saito *et al.* (2007 ▶, 2008 ▶); Puff *et al.* (1989 ▶); Schneider *et al.* (1997 ▶). For theoretical calculations see: Nagase *et al.* (1991 ▶); Gordon *et al.* (1991 ▶); Nguyen *et al.* (1991 ▶); Sandstroem & Ottosson (2005 ▶). For related literature, see: Nagase *et al.* (1988 ▶).
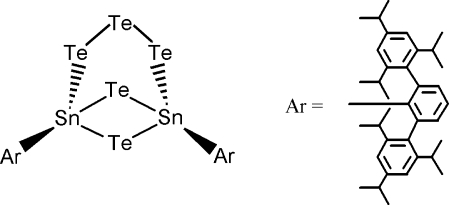


## Experimental

### 

#### Crystal data

[Sn_2_(C_72_H_98_)Te_2_(Te_3_)]
*M* *_r_* = 1838.92
Monoclinic, 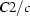

*a* = 24.370 (4) Å
*b* = 11.2673 (19) Å
*c* = 26.620 (4) Å
β = 96.430 (4)°
*V* = 7263 (2) Å^3^
*Z* = 4
Mo *K*α radiation
μ = 2.69 mm^−1^
*T* = 103 K
0.15 × 0.15 × 0.05 mm

#### Data collection

Bruker APEX CCD area-detector diffractometer
Absorption correction: multi-scan *SADABS*; (Sheldrick, 1996 ▶) *T*_min_ = 0.674, *T*_max_ = 0.874
26082 measured reflections
8733 independent reflections
6697 reflections with *I* > 2σ(*I*)
*R*_int_ = 0.052

#### Refinement

*R*[*F* ^2^ > 2σ(*F* ^2^)] = 0.059
*wR*(*F* ^2^) = 0.153
*S* = 1.06
8733 reflections
383 parameters
H-atom parameters constrained
Δρ_max_ = 2.83 e Å^−3^
Δρ_min_ = −1.08 e Å^−3^

### 

Data collection: *SMART* (Bruker, 2000 ▶); cell refinement: *SAINT* (Bruker, 2000 ▶); data reduction: *SAINT*; program(s) used to solve structure: *SHELXS97* (Sheldrick, 2008 ▶); program(s) used to refine structure: *SHELXL97* (Sheldrick, 2008 ▶); molecular graphics: *SHELXTL* (Sheldrick, 2008 ▶); software used to prepare material for publication: *SHELXTL*.

## Supplementary Material

Crystal structure: contains datablocks global, I. DOI: 10.1107/S1600536810025729/fj2324sup1.cif

Structure factors: contains datablocks I. DOI: 10.1107/S1600536810025729/fj2324Isup2.hkl

Additional supplementary materials:  crystallographic information; 3D view; checkCIF report

## Figures and Tables

**Table:** 

Sn1—Te4	2.7353 (7)
Sn1—Te3^i^	2.7617 (14)
Sn1—Te1	2.8383 (15)
Te1—Te2	2.705 (2)
Te2—Te3	2.6792 (18)

**Table:** 

C1—Sn1—Te4	117.69 (14)
C1—Sn1—Te4^i^	122.56 (14)
Te4—Sn1—Te4^i^	96.03 (2)
Te3—Te2—Te1	104.02 (5)

Symmetry code: (i) 
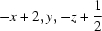
.

**Fig. 1.**
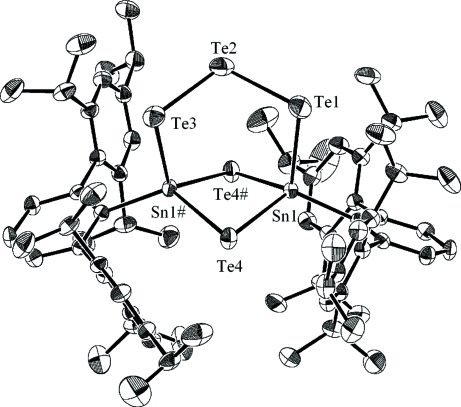
Top view of the molecule of (1) showing the atom-labelling scheme. Displacement ellipsoids are drawn at the 40% probability level. All H atoms and another disorderded part are omitted for clarity.

## supplementary crystallographic information

### Comment

For a few decades much attention has been paid to the chemistry of cage-like compounds containing heavier Groups 14 and 16 elements from the standpoints of their unique structure and reactivity. Among cage-like compounds, the nature of the bridgehead bond of bicyclo[1.1.1]pentanes is of considerable interest because the type of H_2_M_2_X_3_ [1.1.1]propellanes (*M* = Si, Ge, Sn; X = O) is predicted to have a short non-bonded distance between the two bridgehead group 14 atoms (Nagase *et al.*, 1991; Gordon *et al.*, 1991; Nguyen *et al.*, 1991; Sandstroem and Ottosson; 2005). Although no reports on the synthesis of trioxadimetallabicyclo[1.1.1]pentanes of heavier Group 14 elements have so far appeared, trithia- and triselena-derivatives have been relatively well investigated. The synthesis of trithia- and triselenadimetallabicyclo[1.1.1]pentanes was accomplished by the dechalcogenation of the corresponding polythia- and poly-selenadimetalla-bicyclo[k.l.m]alkanes (Yoshida *et al.*, 1992; Ando, Choi *et al.*, 1994; Ando, Kabe *et al.*, 1994; Ando *et al.*, 1995; Choi *et al.*, 1995; Choi *et al.*, 1996; Choi *et al.*, 1997). As for tin analogues, we have recently reported the synthesis, structures and reactions of penta- and tetra-chalcogenadistannabicyclo[k.1.1]alkanes (Saito *et al.*, 2007; Saito *et al.*, 2008). However, no tellurium versions of group 14 [k.l.m]alkanes have been thus far reported. We report herein the first X-ray characterization of the title compound, 1,3-bis[2,6-bis(2,4,6-triisopropylphenyl)phenyl]-2,4,5,6,7-pentatellura-1,3-distannabicyclo[3.1.1]heptane with bulky aryl substituents on the tin atoms.

The X-ray structural analysis reveals that the title compound, 1,3-bis[2,6-bis(2,4,6-triisopropylphenyl)phenyl]-2,4,5,6,7-pentatellura-1,3-distannabicyclo[3.1.1]heptane (1), has a rare tritelluride unit in its cage structure, where one of the six-membered rings has a boat conformation, whereas the other has a chair conformation. The molecule sits over a crystallographic twofold axis, and hence a half moiety of the molecule was refined. The tritelluride moiety has 1: 1 disordered two parts. There have been only four examples of X-ray characterized neutral tritellurides (Sladky *et al.*, 1985, Hamor *et al.*, 1986; Herberhold *et al.*, 1990; Beckmann *et al.*, 2009). The tellurium-tellurium bond), and hence the structures of a tritellurides are of still considerable interest. The bond angle of the central tellurium atom in the tritelluride unit is 104.02 (5) °, similar to those found in the reported neutral tritellurides (93–106 °) (Sladky *et al.*, 1985, Hamor *et al.*, 1986; Herberhold *et al.*, 1990; Beckmann *et al.*, 2009). The tellurium-tellurium bond distances (2.6792 (18) and 2.705 (2) Å) are in the same range as those of the reported neutral tritellurides (2.710–2.776 Å). The ditelluadistannetane ring has a bent structure with the dihedral angles between the Te4—Sn1—Te4# and Te4—Sn1#—Te4# planes of 32.89 (2) °. The tin-tellurim bond distances in the four-membered ring are 2.7353 (7) and 2.7556 (6) Å, similar to those found in ditelluradistannetane rings (2.754–2.771 Å) (Puff *et al.*, 1989; Schneider *et al.*, 1997). The sum of the internal bond angles (C1—Sn1—Te4#, Te4—Sn1—Te4# and C1—Sn1—Te4) around the tin atom is 336.3 °, which remarkably deviates from the ideal *sp*^3^ geometry of 328.5 °.

### Experimental

A mixture of sodium (57.0 mg, 2.48 mmol) and tellurium (157.8 mg, 1.24 mmol) and a catalytic amount of naphthalene (33.1 mg, 0.26 mmol) in THF (3 ml) was heated under reflux for 5.5 h. To the mixture was added a THF (2 ml) solution of 2,6-bis(2,4,6-triisopropylphenyl)phenyltrichlorostannane (170.5 mg, 0.24 mmol) (Saito *et al.*, 2007) at room temperature. The resulting mixture was subjected to gel permeation chromatography to afford the title compound, 1,3-bis[2,6-bis(2,4,6-triisopropylphenyl)phenyl]-2,4,5,6,7-pentatellura-1,3-distannabicyclo[3.1.1]heptane (1) (62.1 mg, 28%).

### Refinement

Hydrogen atoms attached to C(*sp*^3^) and C(*sp*^2^) carbon atoms were treated as riding with C—H distances of 0.96 and 0.93 Å, while all the other atoms were refined anisotropically.

### Crystal data

**Table:**

[Sn_2_(C_72_H_98_)Te_2_(Te_3_)]	*F*(000) = 3560
*M**_r_* = 1838.92	*D*_x_ = 1.682 Mg m^−^^3^
Monoclinic, *C*2/*c*	Mo *K*α radiation, λ = 0.71073 Å
Hall symbol: -C 2yc	Cell parameters from 5522 reflections
*a* = 24.370 (4) Å	θ = 2.2–25.8°
*b* = 11.2673 (19) Å	µ = 2.69 mm^−^^1^
*c* = 26.620 (4) Å	*T* = 103 K
β = 96.430 (4)°	Cubic, red
*V* = 7263 (2) Å^3^	0.15 × 0.15 × 0.05 mm
*Z* = 4	

### Data collection

**Table:**

Bruker APEX CCD area-detector diffractometer	8733 independent reflections
Radiation source: fine-focus sealed tube	6697 reflections with *I* > 2σ(*I*)
graphite	*R*_int_ = 0.052
φ and ω and ω scans	θ_max_ = 28.0°, θ_min_ = 1.5°
Absorption correction: multi-scan *SADABS*; (Sheldrick, 1996)	*h* = −31→32
*T*_min_ = 0.674, *T*_max_ = 0.874	*k* = −14→14
26082 measured reflections	*l* = −35→22

### Refinement

**Table:**

Refinement on *F*^2^	Primary atom site location: structure-invariant direct methods
Least-squares matrix: full	Secondary atom site location: difference Fourier map
*R*[*F*^2^ > 2σ(*F*^2^)] = 0.059	Hydrogen site location: inferred from neighbouring sites
*wR*(*F*^2^) = 0.153	H-atom parameters constrained
*S* = 1.06	*w* = 1/[σ^2^(*F*_o_^2^) + (0.0699*P*)^2^ + 38.0368*P*] where *P* = (*F*_o_^2^ + 2*F*_c_^2^)/3
8733 reflections	(Δ/σ)_max_ = 0.002
383 parameters	Δρ_max_ = 2.83 e Å^−^^3^
0 restraints	Δρ_min_ = −1.08 e Å^−^^3^

### Special details

**Table:**

Geometry. All e.s.d.'s (except the e.s.d. in the dihedral angle between two l.s. planes) are estimated using the full covariance matrix. The cell e.s.d.'s are taken into account individually in the estimation of e.s.d.'s in distances, angles and torsion angles; correlations between e.s.d.'s in cell parameters are only used when they are defined by crystal symmetry. An approximate (isotropic) treatment of cell e.s.d.'s is used for estimating e.s.d.'s involving l.s. planes.
Refinement. Refinement of *F*^2^ against ALL reflections. The weighted *R*-factor *wR* and goodness of fit *S* are based on *F*^2^, conventional *R*-factors *R* are based on *F*, with *F* set to zero for negative *F*^2^. The threshold expression of *F*^2^ > σ(*F*^2^) is used only for calculating *R*-factors(gt) *etc*. and is not relevant to the choice of reflections for refinement. *R*-factors based on *F*^2^ are statistically about twice as large as those based on *F*, and *R*- factors based on ALL data will be even larger.

### Fractional atomic coordinates and isotropic or equivalent isotropic displacement parameters (Å^2^)

**Table:**

	*x*	*y*	*z*	*U*_iso_*/*U*_eq_	Occ. (<1)
Sn1	1.007086 (16)	0.69265 (4)	0.184873 (14)	0.02940 (12)	
C1	1.0110 (2)	0.7739 (5)	0.1112 (2)	0.0260 (11)	
C2	0.9642 (2)	0.7727 (5)	0.0750 (2)	0.0265 (11)	
C3	0.9697 (2)	0.8135 (6)	0.0266 (2)	0.0336 (13)	
H1	0.9387	0.8151	0.0028	0.040*	
C4	1.0197 (2)	0.8516 (6)	0.0128 (2)	0.0354 (13)	
H2	1.0229	0.8749	−0.0202	0.042*	
C5	1.0650 (2)	0.8546 (6)	0.0492 (2)	0.0340 (12)	
H3	1.0987	0.8818	0.0403	0.041*	
C6	1.0615 (2)	0.8182 (5)	0.0981 (2)	0.0271 (11)	
C7	0.9070 (2)	0.7350 (5)	0.0846 (2)	0.0283 (11)	
C8	0.8878 (2)	0.6205 (6)	0.0715 (2)	0.0346 (13)	
C9	0.8325 (2)	0.5928 (6)	0.0734 (2)	0.0372 (13)	
H46	0.8202	0.5169	0.0641	0.045*	
C10	0.7953 (2)	0.6740 (6)	0.0884 (2)	0.0346 (13)	
C11	0.8147 (2)	0.7856 (6)	0.1025 (2)	0.0344 (13)	
H47	0.7902	0.8404	0.1136	0.041*	
C12	0.8699 (2)	0.8198 (5)	0.1008 (2)	0.0303 (12)	
C13	1.1112 (2)	0.8364 (5)	0.1365 (2)	0.0288 (11)	
C14	1.1144 (2)	0.9396 (5)	0.1662 (2)	0.0299 (12)	
C15	1.1620 (2)	0.9601 (5)	0.1993 (2)	0.0350 (13)	
H48	1.1643	1.0287	0.2189	0.042*	
C16	1.2062 (2)	0.8807 (6)	0.2038 (2)	0.0360 (13)	
C17	1.2022 (3)	0.7810 (6)	0.1740 (2)	0.0384 (14)	
H49	1.2316	0.7277	0.1767	0.046*	
C18	1.1559 (2)	0.7561 (5)	0.1399 (2)	0.0318 (12)	
C19	0.9260 (3)	0.5259 (6)	0.0516 (3)	0.0446 (16)	
H4	0.9639	0.5430	0.0661	0.054*	
C20	0.9122 (4)	0.4004 (7)	0.0665 (4)	0.072 (3)	
H5	0.8792	0.3745	0.0465	0.108*	
H6	0.9066	0.3987	0.1017	0.108*	
H7	0.9422	0.3485	0.0609	0.108*	
C21	0.9239 (3)	0.5351 (8)	−0.0063 (3)	0.065 (2)	
H8	0.9431	0.4689	−0.0188	0.097*	
H9	0.9411	0.6076	−0.0150	0.097*	
H10	0.8861	0.5343	−0.0211	0.097*	
C22	0.7339 (2)	0.6469 (7)	0.0878 (3)	0.0444 (16)	
H11	0.7237	0.6643	0.1216	0.053*	
C23	0.7193 (3)	0.5164 (8)	0.0763 (3)	0.061 (2)	
H12	0.7264	0.4980	0.0424	0.091*	
H13	0.6809	0.5034	0.0796	0.091*	
H14	0.7414	0.4662	0.0996	0.091*	
C24	0.6994 (3)	0.7283 (8)	0.0504 (3)	0.058 (2)	
H15	0.7079	0.8097	0.0586	0.088*	
H16	0.6608	0.7142	0.0525	0.088*	
H17	0.7077	0.7119	0.0167	0.088*	
C25	0.8866 (2)	0.9482 (6)	0.1111 (3)	0.0393 (14)	
H18	0.9266	0.9502	0.1205	0.047*	
C26	0.8593 (3)	1.0083 (7)	0.1533 (3)	0.0543 (18)	
H19	0.8206	1.0184	0.1428	0.081*	
H20	0.8760	1.0845	0.1605	0.081*	
H21	0.8642	0.9599	0.1832	0.081*	
C27	0.8735 (4)	1.0224 (7)	0.0632 (3)	0.065 (2)	
H22	0.8907	0.9871	0.0361	0.097*	
H23	0.8874	1.1015	0.0692	0.097*	
H24	0.8343	1.0253	0.0542	0.097*	
C28	1.0677 (3)	1.0297 (6)	0.1614 (3)	0.0415 (15)	
H25	1.0338	0.9877	0.1485	0.050*	
C29	1.0769 (4)	1.1262 (8)	0.1243 (3)	0.065 (2)	
H26	1.0460	1.1795	0.1213	0.098*	
H27	1.0807	1.0916	0.0919	0.098*	
H28	1.1099	1.1691	0.1360	0.098*	
C30	1.0580 (5)	1.0847 (8)	0.2109 (4)	0.084 (3)	
H29	1.0472	1.0244	0.2332	0.127*	
H30	1.0293	1.1431	0.2054	0.127*	
H31	1.0914	1.1219	0.2257	0.127*	
C31	1.2573 (3)	0.9054 (7)	0.2400 (3)	0.0487 (17)	
H32	1.2483	0.9709	0.2619	0.058*	
C32	1.2743 (4)	0.8040 (8)	0.2731 (4)	0.084 (3)	
H33	1.2426	0.7728	0.2871	0.126*	
H34	1.3012	0.8298	0.3000	0.126*	
H35	1.2900	0.7434	0.2538	0.126*	
C33	1.3037 (4)	0.9467 (13)	0.2120 (4)	0.120 (5)	
H36	1.3350	0.9664	0.2357	0.180*	
H37	1.2923	1.0156	0.1923	0.180*	
H38	1.3137	0.8847	0.1900	0.180*	
C34	1.1559 (2)	0.6477 (6)	0.1064 (2)	0.0397 (14)	
H39	1.1186	0.6386	0.0887	0.048*	
C35	1.1966 (4)	0.6647 (9)	0.0658 (3)	0.069 (2)	
H40	1.1864	0.7342	0.0461	0.103*	
H41	1.1951	0.5964	0.0441	0.103*	
H42	1.2335	0.6739	0.0823	0.103*	
C36	1.1702 (3)	0.5341 (7)	0.1362 (3)	0.060 (2)	
H43	1.2066	0.5411	0.1540	0.090*	
H44	1.1692	0.4681	0.1133	0.090*	
H45	1.1439	0.5217	0.1599	0.090*	
Te1	1.00205 (8)	0.44204 (12)	0.17458 (6)	0.0665 (4)	0.50
Te2	1.04546 (5)	0.38902 (9)	0.26993 (4)	0.0605 (3)	0.50
Te3	0.96724 (6)	0.45635 (12)	0.32712 (5)	0.0521 (3)	0.50
Te4	0.916196 (16)	0.73879 (4)	0.233347 (15)	0.04013 (13)	

### Atomic displacement parameters (Å^2^)

**Table:**

	*U*^11^	*U*^22^	*U*^33^	*U*^12^	*U*^13^	*U*^23^
Sn1	0.0337 (2)	0.0306 (2)	0.02381 (19)	0.00283 (16)	0.00285 (14)	−0.00094 (15)
C1	0.021 (2)	0.030 (3)	0.027 (3)	0.001 (2)	0.005 (2)	−0.001 (2)
C2	0.023 (3)	0.029 (3)	0.027 (3)	−0.001 (2)	0.002 (2)	−0.005 (2)
C3	0.026 (3)	0.046 (4)	0.028 (3)	0.001 (2)	−0.002 (2)	0.003 (2)
C4	0.033 (3)	0.052 (4)	0.022 (3)	0.001 (3)	0.006 (2)	0.005 (2)
C5	0.023 (3)	0.041 (3)	0.039 (3)	0.001 (2)	0.008 (2)	0.002 (3)
C6	0.025 (2)	0.032 (3)	0.025 (3)	0.002 (2)	0.001 (2)	−0.002 (2)
C7	0.019 (2)	0.037 (3)	0.027 (3)	−0.003 (2)	−0.003 (2)	−0.001 (2)
C8	0.031 (3)	0.037 (3)	0.036 (3)	−0.004 (2)	0.001 (2)	−0.006 (2)
C9	0.033 (3)	0.040 (3)	0.038 (3)	−0.009 (3)	0.002 (2)	−0.002 (3)
C10	0.026 (3)	0.048 (4)	0.030 (3)	−0.008 (2)	0.004 (2)	0.001 (3)
C11	0.022 (3)	0.049 (4)	0.032 (3)	0.000 (2)	0.004 (2)	−0.001 (3)
C12	0.028 (3)	0.034 (3)	0.030 (3)	−0.002 (2)	0.006 (2)	0.001 (2)
C13	0.022 (2)	0.036 (3)	0.028 (3)	0.002 (2)	0.001 (2)	−0.002 (2)
C14	0.026 (3)	0.030 (3)	0.035 (3)	−0.004 (2)	0.007 (2)	−0.003 (2)
C15	0.037 (3)	0.029 (3)	0.039 (3)	−0.008 (2)	0.005 (2)	−0.007 (2)
C16	0.031 (3)	0.043 (4)	0.032 (3)	−0.006 (3)	−0.005 (2)	0.000 (3)
C17	0.031 (3)	0.038 (3)	0.043 (4)	0.002 (3)	−0.005 (3)	−0.006 (3)
C18	0.027 (3)	0.034 (3)	0.033 (3)	−0.003 (2)	0.000 (2)	−0.005 (2)
C19	0.034 (3)	0.043 (4)	0.056 (4)	−0.001 (3)	0.000 (3)	−0.016 (3)
C20	0.070 (6)	0.047 (5)	0.099 (7)	0.002 (4)	0.004 (5)	−0.020 (5)
C21	0.052 (4)	0.079 (6)	0.064 (5)	−0.004 (4)	0.010 (4)	−0.038 (4)
C22	0.028 (3)	0.064 (5)	0.041 (4)	−0.013 (3)	0.007 (3)	−0.002 (3)
C23	0.046 (4)	0.069 (5)	0.067 (5)	−0.028 (4)	0.009 (4)	−0.001 (4)
C24	0.028 (3)	0.084 (6)	0.063 (5)	−0.007 (3)	0.002 (3)	0.014 (4)
C25	0.027 (3)	0.034 (3)	0.056 (4)	0.000 (2)	0.000 (3)	−0.006 (3)
C26	0.069 (5)	0.044 (4)	0.051 (4)	0.009 (4)	0.010 (4)	−0.008 (3)
C27	0.086 (6)	0.046 (4)	0.068 (5)	−0.010 (4)	0.033 (5)	0.006 (4)
C28	0.028 (3)	0.034 (3)	0.063 (4)	−0.001 (2)	0.008 (3)	−0.006 (3)
C29	0.073 (5)	0.063 (5)	0.062 (5)	0.029 (4)	0.019 (4)	0.013 (4)
C30	0.121 (8)	0.071 (6)	0.071 (6)	0.053 (6)	0.056 (6)	0.016 (5)
C31	0.047 (4)	0.050 (4)	0.044 (4)	−0.008 (3)	−0.019 (3)	−0.006 (3)
C32	0.090 (7)	0.065 (6)	0.083 (7)	−0.009 (5)	−0.054 (6)	0.005 (5)
C33	0.061 (6)	0.193 (15)	0.096 (8)	−0.067 (8)	−0.038 (6)	0.023 (8)
C34	0.032 (3)	0.042 (4)	0.043 (3)	0.008 (3)	−0.008 (3)	−0.013 (3)
C35	0.065 (5)	0.084 (7)	0.058 (5)	0.004 (5)	0.012 (4)	−0.029 (4)
C36	0.066 (5)	0.042 (4)	0.068 (5)	0.019 (4)	−0.012 (4)	−0.012 (4)
Te1	0.0993 (12)	0.0416 (7)	0.0590 (7)	−0.0035 (8)	0.0109 (9)	−0.0131 (5)
Te2	0.0729 (7)	0.0377 (5)	0.0718 (7)	0.0111 (5)	0.0123 (5)	0.0035 (5)
Te3	0.0616 (7)	0.0397 (6)	0.0553 (7)	−0.0085 (6)	0.0081 (6)	0.0060 (5)
Te4	0.0296 (2)	0.0599 (3)	0.0308 (2)	−0.00078 (18)	0.00283 (15)	0.00159 (18)

### Geometric parameters (Å, °)

**Table:**

Sn1—C1	2.175 (5)	C23—H14	0.9600
Sn1—Te4	2.7353 (7)	C24—H15	0.9600
Sn1—Te4^i^	2.7556 (6)	C24—H16	0.9600
Sn1—Te3^i^	2.7617 (14)	C24—H17	0.9600
Sn1—Te1	2.8383 (15)	C25—C26	1.526 (9)
C1—C6	1.408 (7)	C25—C27	1.529 (11)
C1—C2	1.410 (7)	C25—H18	0.9800
C2—C3	1.387 (8)	C26—H19	0.9600
C2—C7	1.506 (7)	C26—H20	0.9600
C3—C4	1.380 (8)	C26—H21	0.9600
C3—H1	0.9300	C27—H22	0.9600
C4—C5	1.385 (8)	C27—H23	0.9600
C4—H2	0.9300	C27—H24	0.9600
C5—C6	1.378 (8)	C28—C30	1.497 (11)
C5—H3	0.9300	C28—C29	1.503 (10)
C6—C13	1.510 (7)	C28—H25	0.9800
C7—C8	1.403 (8)	C29—H26	0.9600
C7—C12	1.416 (8)	C29—H27	0.9600
C8—C9	1.390 (8)	C29—H28	0.9600
C8—C19	1.546 (9)	C30—H29	0.9600
C9—C10	1.380 (9)	C30—H30	0.9600
C9—H46	0.9300	C30—H31	0.9600
C10—C11	1.379 (9)	C31—C32	1.473 (11)
C10—C22	1.525 (8)	C31—C33	1.497 (13)
C11—C12	1.405 (8)	C31—H32	0.9800
C11—H47	0.9300	C32—H33	0.9600
C12—C25	1.520 (8)	C32—H34	0.9600
C13—C14	1.404 (8)	C32—H35	0.9600
C13—C18	1.411 (8)	C33—H36	0.9600
C14—C15	1.394 (8)	C33—H37	0.9600
C14—C28	1.521 (8)	C33—H38	0.9600
C15—C16	1.395 (9)	C34—C36	1.524 (10)
C15—H48	0.9300	C34—C35	1.559 (11)
C16—C17	1.373 (9)	C34—H39	0.9800
C16—C31	1.513 (8)	C35—H40	0.9600
C17—C18	1.396 (8)	C35—H41	0.9600
C17—H49	0.9300	C35—H42	0.9600
C18—C34	1.512 (8)	C36—H43	0.9600
C19—C20	1.516 (11)	C36—H44	0.9600
C19—C21	1.540 (11)	C36—H45	0.9600
C19—H4	0.9800	Te1—Te3^i^	0.7720 (15)
C20—H5	0.9600	Te1—Te2^i^	2.065 (2)
C20—H6	0.9600	Te1—Te2	2.705 (2)
C20—H7	0.9600	Te2—Te1^i^	2.065 (2)
C21—H8	0.9600	Te2—Te2^i^	2.347 (2)
C21—H9	0.9600	Te2—Te3^i^	2.6770 (19)
C21—H10	0.9600	Te2—Te3	2.6792 (18)
C22—C24	1.535 (10)	Te3—Te1^i^	0.7720 (15)
C22—C23	1.536 (11)	Te3—Te2^i^	2.6770 (19)
C22—H11	0.9800	Te3—Sn1^i^	2.7617 (14)
C23—H12	0.9600	Te4—Sn1^i^	2.7556 (6)
C23—H13	0.9600		
			
C1—Sn1—Te4	117.69 (14)	H15—C24—H16	109.5
C1—Sn1—Te4^i^	122.56 (14)	C22—C24—H17	109.5
Te4—Sn1—Te4^i^	96.03 (2)	H15—C24—H17	109.5
C1—Sn1—Te3^i^	105.56 (15)	H16—C24—H17	109.5
Te4—Sn1—Te3^i^	116.34 (3)	C12—C25—C26	115.2 (6)
Te4^i^—Sn1—Te3^i^	97.51 (3)	C12—C25—C27	110.1 (6)
C1—Sn1—Te1	109.72 (15)	C26—C25—C27	107.7 (6)
Te4—Sn1—Te1	101.89 (4)	C12—C25—H18	107.9
Te4^i^—Sn1—Te1	106.39 (4)	C26—C25—H18	107.9
Te3^i^—Sn1—Te1	15.77 (3)	C27—C25—H18	107.9
C6—C1—C2	119.7 (5)	C25—C26—H19	109.5
C6—C1—Sn1	120.1 (4)	C25—C26—H20	109.5
C2—C1—Sn1	120.0 (4)	H19—C26—H20	109.5
C3—C2—C1	118.5 (5)	C25—C26—H21	109.5
C3—C2—C7	116.0 (5)	H19—C26—H21	109.5
C1—C2—C7	125.4 (5)	H20—C26—H21	109.5
C4—C3—C2	122.0 (5)	C25—C27—H22	109.5
C4—C3—H1	119.0	C25—C27—H23	109.5
C2—C3—H1	119.0	H22—C27—H23	109.5
C3—C4—C5	118.8 (5)	C25—C27—H24	109.5
C3—C4—H2	120.6	H22—C27—H24	109.5
C5—C4—H2	120.6	H23—C27—H24	109.5
C6—C5—C4	121.5 (5)	C30—C28—C29	109.2 (6)
C6—C5—H3	119.2	C30—C28—C14	113.1 (6)
C4—C5—H3	119.2	C29—C28—C14	111.9 (5)
C5—C6—C1	119.3 (5)	C30—C28—H25	107.5
C5—C6—C13	117.9 (5)	C29—C28—H25	107.5
C1—C6—C13	122.6 (5)	C14—C28—H25	107.5
C8—C7—C12	119.3 (5)	C28—C29—H26	109.5
C8—C7—C2	120.4 (5)	C28—C29—H27	109.5
C12—C7—C2	119.8 (5)	H26—C29—H27	109.5
C9—C8—C7	119.7 (6)	C28—C29—H28	109.5
C9—C8—C19	118.7 (5)	H26—C29—H28	109.5
C7—C8—C19	121.6 (5)	H27—C29—H28	109.5
C10—C9—C8	122.2 (6)	C28—C30—H29	109.5
C10—C9—H46	118.9	C28—C30—H30	109.5
C8—C9—H46	118.9	H29—C30—H30	109.5
C11—C10—C9	117.9 (5)	C28—C30—H31	109.5
C11—C10—C22	119.4 (6)	H29—C30—H31	109.5
C9—C10—C22	122.7 (6)	H30—C30—H31	109.5
C10—C11—C12	122.7 (6)	C32—C31—C33	111.2 (9)
C10—C11—H47	118.6	C32—C31—C16	113.2 (6)
C12—C11—H47	118.6	C33—C31—C16	110.8 (6)
C11—C12—C7	118.2 (5)	C32—C31—H32	107.1
C11—C12—C25	119.5 (5)	C33—C31—H32	107.1
C7—C12—C25	122.0 (5)	C16—C31—H32	107.1
C14—C13—C18	120.1 (5)	C31—C32—H33	109.5
C14—C13—C6	119.0 (5)	C31—C32—H34	109.5
C18—C13—C6	120.8 (5)	H33—C32—H34	109.5
C15—C14—C13	118.9 (5)	C31—C32—H35	109.5
C15—C14—C28	120.3 (5)	H33—C32—H35	109.5
C13—C14—C28	120.8 (5)	H34—C32—H35	109.5
C14—C15—C16	121.8 (5)	C31—C33—H36	109.5
C14—C15—H48	119.1	C31—C33—H37	109.5
C16—C15—H48	119.1	H36—C33—H37	109.5
C17—C16—C15	118.1 (5)	C31—C33—H38	109.5
C17—C16—C31	121.2 (6)	H36—C33—H38	109.5
C15—C16—C31	120.7 (6)	H37—C33—H38	109.5
C16—C17—C18	122.7 (6)	C18—C34—C36	112.8 (6)
C16—C17—H49	118.7	C18—C34—C35	110.5 (6)
C18—C17—H49	118.7	C36—C34—C35	109.6 (6)
C17—C18—C13	118.4 (5)	C18—C34—H39	107.9
C17—C18—C34	119.5 (5)	C36—C34—H39	107.9
C13—C18—C34	122.1 (5)	C35—C34—H39	107.9
C20—C19—C21	109.9 (6)	C34—C35—H40	109.5
C20—C19—C8	113.2 (6)	C34—C35—H41	109.5
C21—C19—C8	110.1 (6)	H40—C35—H41	109.5
C20—C19—H4	107.8	C34—C35—H42	109.5
C21—C19—H4	107.8	H40—C35—H42	109.5
C8—C19—H4	107.8	H41—C35—H42	109.5
C19—C20—H5	109.5	C34—C36—H43	109.5
C19—C20—H6	109.5	C34—C36—H44	109.5
H5—C20—H6	109.5	H43—C36—H44	109.5
C19—C20—H7	109.5	C34—C36—H45	109.5
H5—C20—H7	109.5	H43—C36—H45	109.5
H6—C20—H7	109.5	H44—C36—H45	109.5
C19—C21—H8	109.5	Te3^i^—Te1—Te2^i^	136.7 (2)
C19—C21—H9	109.5	Te3^i^—Te1—Te2	79.70 (19)
H8—C21—H9	109.5	Te2^i^—Te1—Te2	57.08 (6)
C19—C21—H10	109.5	Te3^i^—Te1—Sn1	76.47 (17)
H8—C21—H10	109.5	Te2^i^—Te1—Sn1	103.91 (6)
H9—C21—H10	109.5	Te2—Te1—Sn1	96.95 (5)
C10—C22—C24	110.7 (5)	Te1^i^—Te2—Te2^i^	75.33 (8)
C10—C22—C23	113.5 (6)	Te1^i^—Te2—Te3^i^	126.51 (6)
C24—C22—C23	110.1 (6)	Te2^i^—Te2—Te3^i^	64.06 (6)
C10—C22—H11	107.4	Te2^i^—Te2—Te3	63.96 (6)
C24—C22—H11	107.4	Te3^i^—Te2—Te3	117.09 (6)
C23—C22—H11	107.4	Te1^i^—Te2—Te1	114.56 (6)
C22—C23—H12	109.5	Te2^i^—Te2—Te1	47.59 (6)
C22—C23—H13	109.5	Te3—Te2—Te1	104.02 (5)
H12—C23—H13	109.5	Te1^i^—Te3—Te2^i^	83.82 (19)
C22—C23—H14	109.5	Te2^i^—Te3—Te2	51.98 (5)
H12—C23—H14	109.5	Te1^i^—Te3—Sn1^i^	87.76 (17)
H13—C23—H14	109.5	Te2^i^—Te3—Sn1^i^	99.49 (5)
C22—C24—H15	109.5	Te2—Te3—Sn1^i^	91.29 (5)
C22—C24—H16	109.5	Sn1—Te4—Sn1^i^	79.82 (2)

Symmetry codes: (i) −*x*+2, *y*, −*z*+1/2.
